# Flavonoid-Based Combination Therapies and Nano-Formulations: An Emerging Frontier in Breast Cancer Treatment

**DOI:** 10.3390/ph18101486

**Published:** 2025-10-02

**Authors:** Priyanka Uniyal, Ansab Akhtar, Ravi Rawat

**Affiliations:** 1 Department of Pharmaceutical Sciences, School of Health Sciences and Technology, University of Petroleum and Energy Studies (UPES), Dehradun 248007, India; upiya25@gmail.com; 2 Neuroscience Center, School of Medicine, LSU Health, New Orleans, LA 70112, USA

**Keywords:** flavonoids, combination therapy, co-delivery, nano-formulation, breast cancer

## Abstract

Cancer has remained a major global health challenge, with around 20 million new cases and 9.7 million fatalities recorded each year. Even though there has been recent progress in therapies such as radiotherapy, chemotherapy, immunotherapy, and gene therapy, cancer remains a major treatment challenge due to late diagnosis and difficulties in therapeutic effectiveness. Flavonoids, a substantial category of naturally occurring polyphenols, have received considerable interest in recent years for their potential involvement in cancer management and prevention, especially concerning breast cancer. These bioactive compounds, abundant in vegetables, fruits, and herbs, exhibit various therapeutic actions, including antioxidant, anti-inflammatory, and antimutagenic effects. The advanced therapeutic potential of flavonoids, when combined with FDA-approved medicines, offers synergistic effects and enhanced clinical results. Additionally, flavonoid-loaded nano-formulations, involving co-delivery systems, are being explored to increase solubility, stability, and bioavailability, enabling targeted delivery to cancer cells while reducing off-target adverse effects. This review examines the role of flavonoids in the prevention and management of breast cancer, focusing on their dietary sources, metabolism, and pharmacokinetic properties. Furthermore, we explore novel strategies, such as combination therapies with FDA-approved drugs and the application of flavonoid-based nanoformulations, which have the potential to enhance therapeutic outcomes. The clinical application of these strategies has the potential to improve breast cancer treatment and create new opportunities for the advancement of flavonoid-based therapies.

## 1. Introduction

Cancer is a serious disease that still lacks an ultimate cure. According to the World Health Organization (WHO), there are 20 million new cancer cases and 9.7 million people dying annually. Cancer has a significant death rate attributable to diagnostic delays and ineffective treatment modalities [1]. The present cancer statistics of 2024 have shown that cancer cases have remained steady in women and there has been a 1.2% drop in cancer cases in males as compared to the prior statistics, but the risk continues to exist [2]. The decline in cancer case numbers is attributed to advancements in several therapeutic approaches, including radiation therapy, chemotherapy, neoadjuvant therapy, immunotherapy, and gene therapy [3].

Flavonoids (taken from the Latin term flavus, indicating yellow, owing to their shade in nature) are a wide category of polyphenols with low molecular weight, usually found in almost all fruits and vegetables [4]. Natural flavonoids represent a family of over 15,000 chemicals that contribute to the colorful pigments of fruits, plants, vegetables, and medicinal herbs, attracting pollinators to support seed and spore germination and facilitating their growth and development. Flavonoids protect plants from diverse biotic and abiotic challenges, functioning as unique UV filters, signaling molecules, and antibacterial agents [5]. In dietary intake, the physicochemical characteristics of flavonoids determine their metabolic process, i.e., their digestion, absorption, and biotransformation [6]. In cancer cells, flavonoids exert multiple biological effects, affecting proliferation through cell cycle arrest, induction of apoptosis and necrosis and displaying antioxidant, anti-inflammatory, anti-mutagenic, and anti-neoplastic activities. Notably, flavonoids impact cell proliferation via a wide variety of processes, and some of them could interact with numerous cellular components such as intracellular proteins, growth regulators, metal enzymes, transcription genes, and genetic material [7,8]. Flavonoids, when used in combination therapy, enhance the effectiveness of anticancer agents by targeting multiple pathways and mitigating drug resistance. They may potentially function synergistically with conventional therapies, improving therapeutic outcomes while minimizing adverse effects. Furthermore, flavonoids help regulate the tumor microenvironment, facilitating comprehensive cancer treatment [9].

Flavonoids contain strong anticancer effects, but their clinical use poses significant limitations. They demonstrate low water solubility, minimal absorption, and quick metabolism. These pharmacokinetic limitations diminish their absorption and limit their therapeutic efficacy in cancer therapy [7]. To overcome these challenges, nanotechnologies have emerged as a viable strategy in cancer therapy. Due to their nanoscale dimensions and extensive surface area, nanoparticles exhibit effective therapeutic drug transport and enhanced infiltration into tumor vasculature that prevents tumor proliferation [10]. Preliminary clinical studies suggest that nanoparticle-mediated drug delivery enhances treatment efficacy and mitigates adverse effects associated with conventional small-molecule chemotherapy. Furthermore, the use of nanoparticles has not been linked to any novel detrimental consequences [11]. Instead, nano-formulations offer several benefits, including biocompatibility, prolonged drug release, protection of medicines from degradation in biological fluids, biodegradability, and targeted delivery to specific areas via simple administration techniques [12]. Research indicates that plant-derived chemicals, whether used alone or in combination with conventional chemotherapy, can be effectively co-loaded onto nanocarriers to enhance their anti-cancer efficacy [13,14]. In recent years, flavonoid-based nanotechnology has garnered considerable interest as a potential approach to combat cancer [15,16].

Several experimental studies highlight the significant potential of flavonoids in managing breast cancer. This review examines their function in breast cancer prevention and treatment, focusing on dietary sources, pharmacokinetic challenges, including bioavailability and metabolism, and their novel applications for combination therapies and nanoformulation delivery systems. Previous studies have mainly emphasised the anticancer potential of flavonoids or the function of nano-formulations as drug delivery methods. In comparison, this review offers a concentrated discussion on flavonoid-based combination therapy and their nano-formulations in breast cancer. It gives a comparative viewpoint that has not been discussed in existing literature.

## 2. Flavonoids as Natural Therapeutics: Mechanisms and Applications

### 2.1. Structural Insights into the Chemistry of Flavonoids

**Figure 1:**
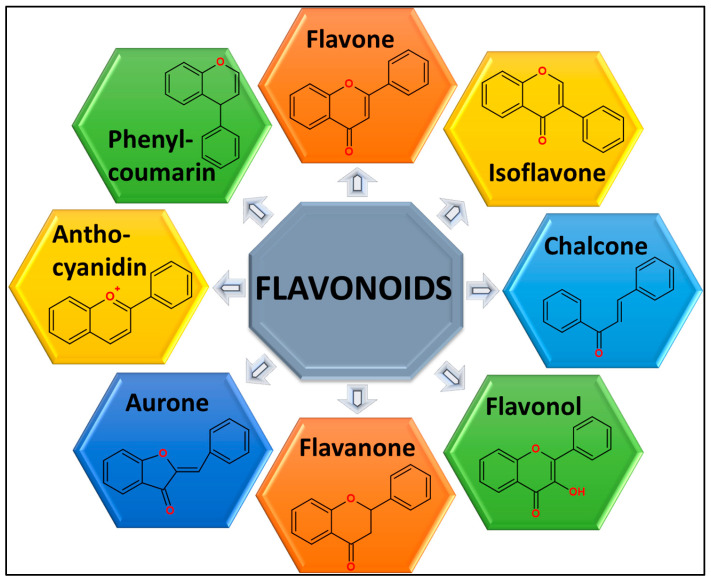
Classification of flavonoids and their chemical structures. Adapted from Vachetta et al. [22] under the Creative Commons CC BY 4.0 license.

Flavonoids are an important class of polyphenolic substances of plant-generated secondary metabolites that contribute to the physiological properties of plant-based products [17]. Flavonoids constitute the primary substances found in foods derived from plants, including vegetables, fruits, nuts, legumes, and grains, while tea and wine serve as extensive sources of consumable flavonoids. Green vegetables, onions, berries, cherries, apples, soybeans, and citrus fruits are considered rich sources of dietary flavonoids [18]. Flavonoids are structurally made up of a C6-C3-C6 flavan backbone, which is made up of 15 carbon atoms organized in two phenyl rings (A and B) joined by a heterocyclic pyran ring (C). Flavonoids are divided into subgroups, including flavones, flavanones, flavonols, flavanols, flavanonols, isoflavones, and anthocyanidins, according to their ring structure and level of saturation [19]. While differences within a subtype result from different substitutions on rings A and B, these subtypes vary in the oxidation state and alteration pattern of ring C [20]. In dietary sources, flavonoids are mostly found as methylated derivatives, glycones, or glycosides. For example, benzene, which may exist in a dihydro form, is fused to an α-pyrone molecule with a six-membered heterocyclic ring in flavonols and flavanones. While the benzenoid group is located at the second carbon in flavonoids, it moves to the third position in isoflavonoids. The presence of a double bond at the C2-C3 location and a hydroxyl group at the third position in flavonols is a crucial structural difference between them and flavanones [21] (Figure 1).

### 2.2. Nature’s Reservoir: Sources of Flavonoids

Flavonoids are commonly present in tomatoes, mulberries, Amazon grapes, apples, and citrus fruits [23,24,25,26,27]. These flavonoid-rich fruits and vegetables possess a beneficial impact against breast cancer and exhibit chemopreventive potential. From the consumption of foods, flavonoids bind with proteins, showing the possibility of utilizing synthetic analogs as anti-cancer treatments [28]. Tomatoes are an excellent source of flavonoids and other phytochemicals, including phenolic compounds, carotenoids, vitamins, and tomatins (glycoalkaloids), which are beneficial for preventing malignancies and treating chronic degenerative conditions [29,30]. Several flavonoids in tomatoes exhibit anti-proliferative capabilities by promoting apoptosis in various types of cancer cells [31].

Additionally, quercetin-3-β-D-glucoside, isolated from tomatoes, is a powerful, stable, and non-toxic molecule with anti-proliferative effects on cancer cells [32,33]. Among them, quercetin and related flavonoids are particularly effective in preventing the development of breast cancer in MCF7 cell lines, resulting in low cytotoxicity [34]. Furthermore, polyphenols and flavonoids obtained from tomatoes interact with the regulation of multiple transcription factors. Their actions also alter the phenylpropanoid biosynthesis pathway, as phenylalanine is transformed into trans-cinnamic acid by phenylalanine ammonia-lyase (PAL). It is further accelerated by cinnamic acid 4-hydroxylase (C4H) to yield *p*-coumaric acid. This mechanism is related to the strategic accumulation of polyphenols and the use of phenylalanine under the shikimate pathway [35,36].

Mulberries, a flavonoid-rich fruit, have been extensively utilized in indigenous practices for their antioxidant effects [37]. Mulberries, a food rich in flavonoids, have been historically used in folk medicine due to their antioxidant properties. Polyphenolic substances found in mulberries, including isoquercitrin, chlorogenic acid, quercetin, astragalin, and kaempferol, showed efficacy in mitigating inflammation associated with obesity and type 2 diabetes [38]. Mulberries have anti-cancer properties against breast cancer cells by inducing apoptosis via pathways associated with PI3K, tumor protein p53, c-Jun N-terminal kinase, and nuclear factor-kappaB (NF-κB) [39,40]. Furthermore, chlorogenic acid, derived from mulberry leaves, is shown to inhibit steatohepatitis by alleviating oxidative stress [41]. It enhances the viability of HepG2 cells by modulating the Nrf-2 signaling system. It decreases inflammation by lowering the production of pro-inflammatory markers such as TNF-α, interleukin 6 (IL-6), inducible nitric oxide synthase (iNOS), and NF-κB [42]. Amazon grapes, which contain phenolic compounds, are reported to enhance the activity of several sulfur-oxidizing enzymes, including glutathione and superoxide dismutase. Such compounds also interact with the production and growth of catalase, antioxidant enzymes, and detoxification [43,44,45]. Amazon grapes are a valuable source of polyphenolic compounds, which exhibit significant disease-preventing effects and have been found beneficial for human health. These compounds give protection to protein and DNA, prevent poor iron activity, and inhibit enzymes.

**Figure 2:**
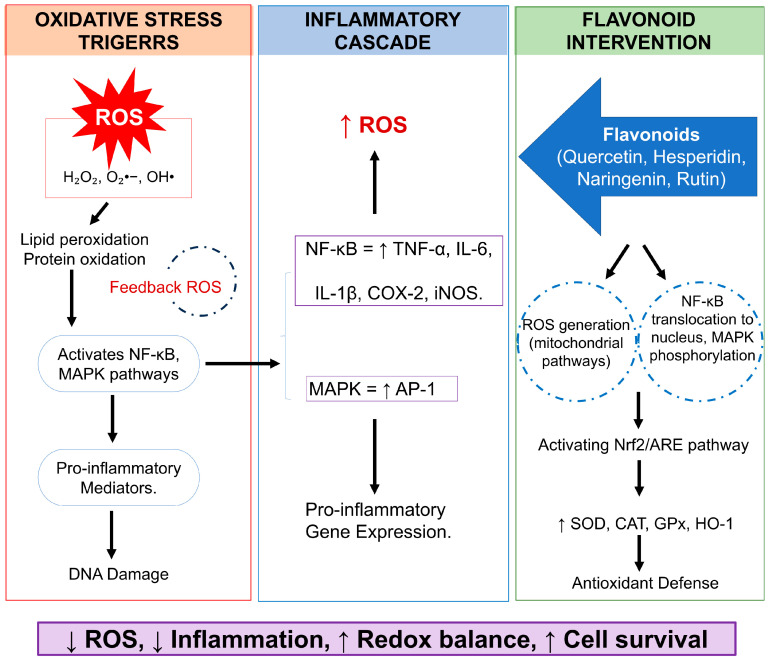
Mechanistic representation of flavonoids attenuating oxidative stress and inflammation.

Furthermore, Amazon grapes have been found to suppress the in vitro activities of tyrosinase, acetylcholinesterase (AChE), and α-amylase, indicating their potential as a preventative therapy for several disorders [46]. The cancer prevention advantages of Amazon grapes are attributed to flavonoids, as well as constituents such as quercetin, catechins, kaempferol, epicatechins, resveratrol, and anthocyanins, which contribute to their chemo-preventive and anti-proliferative activities [47]. Apples are an important source of bioactive components, such as flavonoids, terpenoids, phenols, and carotenoids, which are commonly recognized for their ability to reduce cancer risk [48,49]. These fruits, rich in flavonoids, have been scientifically proven to possess anti-inflammatory, antioxidant, and anticancer properties (Figure 2). The polyphenolic compounds in apples, which include epicatechin, chlorogenic acid, coumaric acid, caffeic acid, quercetin, phlorizin, quercetin-3-glucoside, and phloretin, are significantly beneficial in minimizing the risk of diabetes, cardiovascular diseases, and cancer due to their phytochemical properties [50]. Additionally, quercetin, a flavonoid found in apples, exhibits significant antioxidant properties attributed to its flavonoid, polyphenol, and phytochemical components [51]. The concentration of these active phytochemicals is influenced by harvest timing, with potential degradation during storage [52].

Nobiletin, important for alterations in flavonoid concentration and antioxidant solubility, is reduced as citrus fruits mature and develop. This reduction has been observed in several biologically active compounds present in citrus fruits [53]. Polyphenols are present in various parts of citrus fruits, such as the peels, skin, pulp membrane, seeds, and juice. Such flavonoids produced from citrus have anti-fungal, anti-viral, and anti-bacterial properties [54]. Increased concentrations of flavonoids are mainly found in various varieties of grapefruit and orange, whereas fruits such as mandarin orange, sweet orange, and lemon possess comparatively lower concentrations. In several Asian nations, citrus fruits are frequently used for their antioxidant properties, typically due to the hesperidin that occurs in mandarins, oranges, and grapefruits [54,55].

Furthermore, the flavonoid glycosides present in citrus plants have been identified for their ability to mitigate free radical activity by interacting with hydrogen peroxide, hence protecting against cellular damage. These flavonoids are essential for capillary protection, anti-cancer activity, the mitigation of leg edema, and the alleviation of symptoms associated with hemophilia [56,57]. Citrus fruits, especially lemon, pomelo, mandarin, lime, and grapefruit, are highly abundant in flavonoids, mostly found in their peels. The flavonoids found in citrus peels have potent anti-cancer actions, owing to their free radical scavenging capabilities [58]. The therapeutic efficacy of specific flavonoids present in fruits depends on their mechanism of action and bioavailability, highlighting the need for further pharmacological research and epidemiological studies to elucidate their benefits in cancer therapy [59].

### 2.3. Overcoming the Challenges: Bioavailability and Metabolism

A significant difficulty with flavonoids is their limited bioavailability, which is essential for assuring their efficacy. The approach of bioavailability involves many phases, including liberation, absorption, distribution, metabolism, and elimination (LADME). Several factors, including molecular mass, chemical nature, esterification, glycosylation, and botanical source, influence the uptake of dietary flavonoids [60]. Flavonoids are primarily absorbed due to their solubility and permeability. Moreover, due to their significantly lower molecular weight and lipophilicity, flavonoid aglycones may readily cross the intestinal epithelium. However, flavonoid glycosides have exhibited reduced permeability, likely due to their increased hydrophilicity and higher molecular mass. Hydrophilic aglycone flavonoid glucosides are carried into the epithelium of the small intestine through the Na^+^-dependent glucose cotransporter. They undergo hydrolysis by cytosolic β-glucosidase or lactase-phlorizin hydrolase, a glucosidase located in the brush border membrane of the small intestine. Aglycones are absorbed through the small intestine following hydrolysis, while complex glycosides are transported to the colon for hydrolysis by bacterial enzymes, releasing flavonoid aglycones [61]. Several studies have evaluated the absorption kinetics of naringenin and its glycosides in rats. It has been shown that the absorption kinetics of naringin and naringenin were identical, but naringin showed a delay in absorption in the intestine, which causes decreased bioavailability [62].

Furthermore, after administering naringin, no glucoside was observed in the cecum, indicating successful absorption and bioavailability related to its glycosidic form [63]. Besides, research on the quercetin bioavailability and its glycosidic form upon oral administration to rats has suggested that the aglycone part possesses lower bioavailability (2.0%) in comparison to the quercetin-3-*O*-maltoside and quercetin-3-*O*-glucoside, which produced 30% and 12% bioavailability, respectively [64]. Furthermore, several flavonoid glycosides, such as puerarin 7-*O*-isomaltoside and puerarin 7-*O*-glucoside, have been reported to have increased plasma concentrations and prolonged blood residence times compared to the aglycone upon intravenous dosing [65]. A pharmacokinetic study involving a single dose was performed on 10 healthy participants under 50 years old to evaluate the 24-h absorption and elimination of phenolic acids, flavonoids, and proanthocyanidins with a cranberry juice formulation comprising 54% liquid. The juice predominantly contained anthocyanins, specifically 3-arabinoside, 3-galactoside, cyanidin-3-arabinoside, and peonidin-3-galactoside, along with flavonols like quercetin and hyperoside. The average concentration of phenolic compounds, comprising flavonols, phenolic acids, and flavanols, observed in plasma reached 34.2 g/mL at 8–10 h, with some concentration of quercetin, protocatechuic acid, and vanillic acid [66]. The delivery and local circulation of nutritional flavonoids are affected by the binding ability to plasma proteins, including hemoglobin [67]. Methylated flavonoids have a protein affinity that is 2 to 16 times superior to that of non-methylated flavonoids, principally attributable to enhanced hydrophobic contact with human plasma albumin and ovalbumen [68]. During absorption, flavonoids are considered xenobiotics in the body and are rapidly eliminated from the circulation by hepatic mechanisms, including methylation, sulfation, and glucuronidation.

Furthermore, they may be metabolized into lighter phenolic molecules [69]. Such metabolic processes seem to be as effective as P450-associated oxidation and potentially contribute to the metabolism of certain dietary components [70]. The colon is essential for the biological absorption and metabolic processes of dietary phenolic and polyphenolic substances, releasing distinct catabolites that affect the gut microbiome [71]. A significant quantity of flavonoid derivatives in urine signifies extensive metabolism of such chemicals from the colonic microflora [72]. Yang et al. (2022) indicated that the urinary recovery percentages of the glucoside or rhamnoglucoside forms of naringenin were smaller (31% and 14%, respectively) compared with the aglycone form (14%), claiming that more than 70% of the flavonoid is potentially available to produce specific physiological effects [63].

## 3. Epidemiological Studies: Role of Flavonoids in Breast Cancer Prevention

One of the case–control studies carried out by Ingram et al. suggested a correlation between phytoestrogen intake and breast cancer outcome, showing a notable decrease in breast cancer risk among women with higher consumption of phytoestrogens, specifically the lignan enterolactone and isoflavonic phytoestrogen equol. The consumption of phytoestrogens was estimated using urine excretion examination in 144 specimens. The results indicated that increased consumption of these substances may have a promising role in preventing breast cancer [73]. An examination of 250 urine specimens of Chinese women in Shanghai showed that increased excretion values for total lignans and isoflavonoids were linked to a lower chance of breast cancer. The modified risk ratio for women exhibiting higher excretion levels of both phytoestrogen types, compared to those with reduced excretion, was 0.28 (95% CI: 0.15–0.50). This substantiated the claim that elevated phytoestrogen consumption may have a preventive effect against breast cancer [74]. Another case–control study conducted in New York provides further evidence for this association by analyzing flavonoid consumption among 1434 breast cancer patients and 1440 controls. Participants completed a meal frequency questionnaire to assess their dietary habits over the past year. The findings indicated a significantly decreased risk of cancer in subjects consuming higher levels of flavonoids, including lignans, flavones, flavonols, and flavan-3-ols, especially in postmenopausal women [75]. Similar case–control research done by Tu et al. identified a strong and inverse correlation between flavone consumption and breast cancer development in a group of 820 breast cancer patients and 1548 controls. This research indicated a 13% decrease in breast cancer incidence per standard deviation (0.5 mg/day) with a rise in flavone consumption. Inverse relationships were also identified for anthocyanidins, flavan-3-ols, and flavonols [76].

In U.S.-based cohort research comprising 56,630 postmenopausal women, a meta-analysis by Ying Wang et al. evaluated the association between seven subtypes of dietary flavonoids and the possibility of estrogen receptor (ER)-related invasive postmenopausal breast cancer. During the period from inception till August 2021, a total of 2116 incidences of invasive breast cancer were identified. The research showed a moderate inverse correlation between flavone consumption and the total probability of developing breast cancer and a connection between flavan-3-ol administration and ER-negative rates of breast cancer [77]. Multiple meta-analyses have also shown the connection between soy isoflavone consumption and lower breast cancer incidence [78,79,80]. Additionally, research by Hui et al., which analyzed 9513 incidents and 181,906 controls across six prospective cohort studies and six case–control analyses, found that consumption of flavonols and flavones, rather than other flavonoid subtypes, was associated with a decreased breast cancer risk among postmenopausal women [81]. Furthermore, another meta-analysis examining five cohort experiments comprising 11,206 patients revealed that soy food intake may be associated with lower recurrence and mortality rates, specifically in individuals with ER^+^/PR^+^, ER-negative, and postmenopausal breast tumors [82].

## 4. Unlocking the Chemotherapeutic Potential of Flavonoids in Breast Cancer Treatment

Flavonoids and polyphenolic substances are considered promising agents in the therapeutic arena of breast cancer treatment based on their potential to regulate various physiological processes involved in cancer development [83,84]. These naturally occurring compounds in teas, vegetables, fruits, and herbs provide a wide range of biological actions, including antioxidant, anti-inflammatory, and anticancer effects [85,86] (Table 1). Quercetin, an interesting flavonoid, is commonly found in apples, onions, and leafy green plants. It has been thoroughly investigated for its anticancer potential [87,88,89]. In this context, quercetin has been depicted to suppress cell growth in breast cancer cell lines, such as MCF-7 and MDA-MB-231, by activating multiple signaling pathways. Studies have demonstrated that quercetin promotes apoptosis by modulating the Bcl2/Bax ratio and inhibiting the PI3K/AKT pathway [90,91,92]. In triple-negative breast cancer (TNBC) cells, quercetin reduces the expression of fatty acid synthase and β-catenin, suggesting its ability to inhibit metastasis by modulating epithelial–mesenchymal transition (EMT) signals [93,94].

Additionally, quercetin synergizes with chemotherapy drugs, including tamoxifen and doxorubicin, improving their potency [95,96]. Luteolin is another flavonoid exhibiting significant anti-cancer properties. It has been identified for suppressing tumor proliferation and triggering apoptosis in breast cancer cells [97,98]. Research indicates that luteolin, when combined with lapatinib, enhances the susceptibility of HER2-positive breast cancer cells to therapy, thereby increasing the effectiveness of lapatinib [99]. Furthermore, luteolin inhibits angiogenesis by hindering VEGF, thereby enhancing its efficacy as an anticancer agent [100]. Likewise, puerarin, extracted from the kudzu plant, has gained interest due to its phytoestrogenic properties [101,102]. It increases apoptotic levels in MCF-7 cell lines and has been shown to reduce cell adhesion and movement. Although its clinical use is limited by inadequate solubility, new technologies, especially nanotechnology, have helped enhance its bioavailability, thereby offering an alternative to conventional treatments [103].

Additionally, apigenin is found in various vegetables and fruits and has demonstrated the ability to disrupt cell cycle development in breast cancer cells. The mechanism comprises the modulation of critical signaling pathways, the inhibition of IL-6, and the prevention of tumorigenesis [104,105]. Apigenin has also been demonstrated to augment the efficacy and protective properties of doxorubicin against chemotherapy-induced toxicity [106,107]. A flavanone, specifically isoliquiritigenin, extracted from liquorice, has the potential to induce apoptosis and inhibit proliferation in breast cancer cells [108]. It has also been demonstrated to inhibit the PI3K/AKT pathway, resulting in decreased levels of cyclins and inhibition of cancer cell growth [109]. Structural variations of isoliquiritigenin have yielded derivatives with enhanced bioactivity, underscoring its potential as a therapeutic agent [110].

Furthermore, curcumin, extracted from turmeric, is regarded as one of the most thoroughly researched plant-derived chemicals for the management of breast cancer. It demonstrates a wide array of anticancer effects, encompassing the suppression of cell growth, cancer metastases, and angiogenesis [111,112]. Curcumin also enhances the effectiveness of chemotherapy, including metformin, by promoting apoptosis and reducing tumor proliferation [113]. Considering its intriguing effects, the limited bioavailability of curcumin has stimulated research into various methods of delivery and structural modifications to enhance its therapeutic efficacy [114,115].

**Table 1:** Therapeutic effects of flavonoids in breast cancer treatment. Adapted from Vachetta et al. [22] under the Creative Commons CC BY 4.0 license.

Flavonoid	In Vitro/In Vivo	Breast Cancer Cell Lines	Effects	References
Hesperidin; Apigenin; Quercetin (Propolis)	In vitro	MCF-7	Accumulation in G0/G1 phase, cell cycle, proliferation, apoptosis	[116,117]
Quercetin	In vitro	MCF-7Ca/TAM-R	Increase in ERα and inhibition of HER2	[118]
	Nude/MCF-7	MCF-7	Inhibition of von Willebrand Factor (vWF); suppression of calcineurin activity; tumor microvessel density modulation; decrease in VEGF/VEGFR2 signaling and NFAT activation.	[119]
Hesperidin	In vitro	MCF-7	Increase apoptosis via G0/G1 phase arrest, caspase-3 and caspase-9 upregulation, increase BAX activation, and inhibit BCL-2 expression.	[120]
Luteolin	In vitro	MCF-7	Increase apoptosis via G0/G1 phase arrest, caspase-8 and caspase-9 upregulation and inhibit BCL-2, pAKT, pIGF-1R, Erα expression.	[120]
	Nude/T47D	T47D	VEGF secretion and mRNA expression, tumor microvessel density, tumor-specific VEGF expression, and BAX levels	[121,122]
Nobiletin	In vitro	MCF-7	Increase in CYP1 enzyme activity, elevation of CYP1A1 protein expression, upregulation of CYP1B1 mRNA levels, and G1 cell cycle arrest.	[123]
Eupatorin	In vitro	MCF-7	Increased apoptosis in G2/M phase; enhanced activity of BAX, caspase-9, and caspase-8; modulation of RAF-1 and inhibition of VEGFA, BCL2L11, CHK1, CHK2, HIF1A, and AKT	[124]
Xanthohumol	In vitro	MCF-7	Enhance apoptosis during G1 phase arrest while reducing the levels of pAKT (S473); pMAPK (T202/Y204); and phosphorylated ERα at multiple sites (S104/S106, S118, S167, S305, Y537).	[125]
Silibinin	In vitro	MCF-7, T47D	Enhanced BAX expression; mitochondrial cytochrome c release; nuclear translocation of AIF; induction of autophagy; activation of caspase-8, and reduced BCL-2 expression; along with modulation of ERα and ERβ activity.	[126,127]
Kaempferol	Nude/MCF-7	MCF-7	Increase cleaved PARP; BAX and downregulation of BCL-2, pAKT, pMEK1/2, pERK1/2 and pIRS-1.	[128,129]
Chalcone; Licochalcone A	In vitro	MCF-7	Induce plasma membrane damage; BAX upregulation; cleaved PARP, and CIDEA; downregulate G2/M and S cell cycle phases, cyclin-D1, and BCL-2.	[130]
LW-214 (flavone)	Nude/MCF-7	MCF-7	Enhance BAX expression; cleaved PARP, caspase-9, ROS generation; mitochondrial cytochrome c release; nuclear translocation of AIF, pJNK, and pASK1 levels; reducing BCL-2 and TRX-1 expression.	[131]
NSC 686288 (flavone)	In vitro	MCF-7	Increase cleaved PARP, caspase-9, and ROS levels while decreasing AhR signaling along with the expression of CYP1A1 and CYP1B1.	[132]
2′-Nitroflavone	In vitro	MCF-7	Cytotoxicity	[133]
Pentamethoxylated-flavone	In vitro	MCF-7	Alters the expression of the BCL-2 protein and promotes cell death.	[134]
Puerarin	In vitro	MCF-7, LPS	Decrease NF-κB p65, MMP-9; MMP-2; CCR7; CXCR4; VCAM-1; ICAM-1; TNFα; IL-6; pNF-κB p65; pIκBα; pERK1/2 Downregulate NF-κB MMP-9, p65, CCR7, MMP-2, VCAM-1, TNFα, CXCR4, ICAM-1, pIκBα, p65, IL-6, pNF-κB and pERK1/2.	[135]
Calycosin	In vitro	MCF-7, T47D	Decrease FOXP3; MMP-9; VEGF, MMP-9;	[136]
Orientin	In vitro	MCF-7, TPA	Reduce IL-8 levels; PKCα membrane translocation; pERK activation, and nuclear translocation of c-JUN, c-FOS, and STAT3.	[137]
Corylin	In vitro	MCF-7	Increase miR-34c and decrease LINC00963 mRNAMMP-9; cytotoxicity	[138]
Hinokiflavone	In vitro	MCF-7	Decrease MMP-9; cytotoxicity	[139]
3,6-Dihydroxy flavone	In vitro	MCF-7	Upregulation of E-cadherin with downregulation of SNAIL, TWIST, SLUG, N-cadherin, NOTCH1, and NICD.	[140]
LFG-500 (Flavone)	MMTV-PyMT transgenic mice	MCF-7	Upregulation ZO-1; E-cadherin; pYAP; pMST1/2; pLATS1/2 and reduction in N-cadherin; vimentin; SLUG; SNAIL; YAP; ILK	[141]
Hispidulin	In vitro	MCF-7	Upregulation of E-cadherin and downregulation of occludin; pSMAD2/3	[142]
Calycosin	In vitro	Nude/T47D	Upregulation of E-cadherin and downregulation of N-cadherin, vimentin, CD147, MMP-2, MMP-9, and BATF.	[143]
2′-Hydroxy flavanone	Nude/MCF-7	MCF-7	Upregulation of E-cadherin and downregulation of vimentin, along with modulation of RLIP76 and ERα expression.	[144,145]
Kaempferol	In vitro	MCF-7	Upregulation of E-cadherin and downregulation of N-cadherin, SNAIL, SLUG, cathepsin D, MMP-9, and MMP-2.	[128,129]
Wogonoside	Nude/MCF-7	MCF-7	Downregulation of VEGF expression; inhibition of VEGF promoter activity; suppression of endothelial cell (EC) migration; reduction in EC invasion, and impairment of tubulogenesis.	[146]
Jaceidin	Swiss albino /Ehrlich Ascites Carcinoma cells	MCF-7	serum VEGF	[147]

There are numerous mechanisms of action of flavonoids that demonstrate their therapeutic value in breast cancer therapy. Particularly, their capacity to control the cell cycle and promote apoptosis generates interesting credentials. Some of the compounds, including luteolin, quercetin, and hesperidin, promote G0/G1 phase arrest and downregulate anti-apoptotic proteins, such as BCL-2, while upregulating pro-apoptotic markers, including BAX, caspase-3, and caspase-9 [116,117,120,124,126,127]. These outcomes show their ability to promote programmed cell death and inhibit the growth of cancer cells. Flavonoids also display potent anti-angiogenic and anti-metastatic effects. Kaempferol and wogonoside, for this reason, inhibit VEGF expression, which in turn decreases endothelial cell migration and tumor microvessel density [128,129,146]. Hispidulin and kaempferol are two flavonoids that prevent the epithelial–mesenchymal transition (EMT) by reducing the levels of N-cadherin, SNAIL, and SLUG and increasing E-cadherin [142,148]. Their ability to inhibit both angiogenesis and metastasis demonstrates their diverse function in preventing the growth of tumours.

Furthermore, certain flavonoids, including quercetin and nobiletin, target specific pathways, such as tamoxifen resistance or HER2 suppression, to overcome treatment resistance in breast cancer [123]. Others, such as LW-214 and NSC 686288, enhance apoptosis without harming healthy tissues by specifically generating reactive oxygen species (ROS) in cancer cells [131,132]. These results highlight how flavonoids can be utilized to address various cancer hallmarks, making them beneficial substances for potential future treatment approaches.

**Figure 3:**
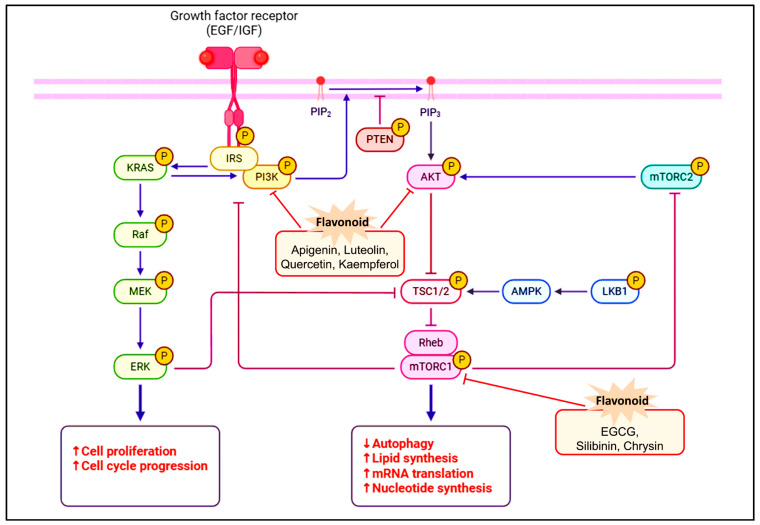
Molecular Targets of Flavonoids Within the PI3K/AKT/mTOR Pathway in Breast Cancer Cells.

Moreover, polyphenols, a diverse category of plant compounds characterized by numerous phenolic hydroxyl groups, are recognized for their unique chemical and biological properties. Polyphenols, including tannins, flavonoids, and phenolic acids, are prevalent in plant-based foods and offer various health benefits. They are thought to significantly contribute to the alleviation of oxidative stress, which is directly associated with cancer development [149,150,151,152]. Epigallocatechin gallate (EGCG), a prominent polyphenol in green tea, demonstrates significant anticancer efficacy. EGCG has been shown to have the ability to regulate oxidative damage, inflammatory reactions, and the growth of cancer cells [153]. It inhibits the development of cell cycles by blocking cells in the G1/S or G2/M phases, thereby successfully decreasing in vivo tumor proliferation [154]. EGCG exhibits a notable anti-angiogenic effect by inhibiting the induction of NF-κB and HIF-1α, while concurrently reducing VEGF expression, thereby inhibiting the generation of new blood vessels essential for tumour proliferation [155,156]. Another non-flavonoid polyphenol is resveratrol, which can be obtained from peanuts, red wine, and grapes. It is well-known for its capacity to prevent the growth of cancer cells and its anti-inflammatory properties [157,158]. Resveratrol enables breast cancer cells to undergo apoptosis and increases the efficacy of chemotherapeutic drugs. It also enhances the effectiveness of traditional treatments by targeting several key points implicated in cancer signaling pathways [159,160,161]. Another class of flavonoid compounds, called anthocyanins, which give colors to various fruits and vegetables, also exhibit strong anti-cancer effects. According to study findings, anthocyanins, like cyanidin-3-glucoside, may prevent the growth of breast cancer cells by causing apoptosis and inhibiting cyclin-dependent kinases [162,163,164]. They improve antioxidant capacity and alter important signaling pathways, like the PI3K/AKT pathway [165,166,167] (Figure 3). A flavonoid present in many fruits and vegetables, kaempferol has proven anti-cancer properties. Kaempferol, through processes like cell cycle arrest and ER suppression, prevents cell division and triggers apoptosis [148,149,168,169]. Interestingly, the establishment of nanosuspensions has improved the therapeutic efficacy of kaempferol by addressing its poor bioavailability [170,171]. Some of the compounds and their derivatives have undergone evaluation in clinical trials for the management of breast cancer (Table 2).

Clinical research exploring flavonoids and their combinations with anticancer agents indicates their therapeutic efficacy in breast cancer management. For example, genistein has been studied for its potential in preventing breast carcinoma and as an adjunct to gemcitabine in metastatic breast cancer [172]. AFP464, a derivative of aminoflavone, has been explored in estrogen receptor-positive breast cancer and solid tumours that exhibit resistance to conventional therapy [173]. ME-344, a synthetic flavonoid-like compound, has anti-angiogenic characteristics and modifies mitochondrial metabolism in HER2-negative breast cancer [174]. Extracts from plants, such as watercress, have been evaluated for their effects on chemotherapy results and the mitigation of DNA damage [175]. *S*-equol, an ERβ agonist, is being studied for its effectiveness in triple-negative breast cancer, highlighting the increasing focus on flavonoid-based approaches for targeted breast cancer treatment [176].

Recent trends in intellectual property emphasise the translational potential of flavonoid-based combination treatments for breast cancer. A number of patents have been published and granted, focused on the combination of flavonoids with kinase inhibitors, chemotherapeutic drugs, CDK inhibitors, and radiotherapy. These patents highlight various dosage forms, such as oral, intravenous, and topical preparations, to improve therapeutic potential and reduce toxicity. These results highlight the increasing acknowledgement of flavonoids as significant adjuvants to recent cancer treatment and provide perspectives on potential clinical and commercial uses (Table 3).

## 5. Synergizing Flavonoids with Synthetic Drugs in Combination Therapy

Natural products originating from the basic chemical constituents of plant material comprise quinonoids, alkaloids, flavonoids, essential oils, polysaccharides, coumarins, terpenoids, and saponins. Multiple studies have demonstrated that plant-derived natural products possess multiple physiological and therapeutic effects, including anti-inflammatory, neuroprotective, antioxidant, antiviral, and cardioprotective effects [177,178,179]. These natural substances are obtained from medicinal herbs globally and are very commonly studied as potential therapeutic interventions. Multiple in vitro and in vivo tests, including some clinical trials, have shown the chemopreventive and anticarcinogenic functions of natural substances against multiple cancers [180,181,182,183]. Intracellular mechanisms linked to tumour formation, such as tumour growth, including cell proliferation, DNA repair, cell differentiation, carcinogen metabolism, apoptosis, angiogenesis, and progression, are possibly linked to the pathways generating the anti-cancer actions [48,184]. Scientific studies on herbal substances against various cancer variants, including lung, breast, and prostate cancer, continue to be explored. As compared to the above-mentioned three kinds of cancers, significantly, a lot more studies have been conducted on breast cancer concerning herbal compounds. Several natural substances exhibit anti-cancer properties for breast cancer cell lines, offering another possibility of treatment for this specific type of cancer. Natural remedies used in combination with chemotherapy medicines may exhibit synergistic or additive effects [185,186]. The statistical concept of therapeutic synergies can be analysed using the combination index (CI), a concept of Chou–Talalay, which proposes whether a combination has an additive effect (CI = 1), synergistic effect (CI < 1), or antagonistic effect (CI > 1) [187]. Moreover, the harmful impact decreased and the drug resistance improved in the case of combination treatments.

Several in vitro and in vivo tests have examined the combined benefits of flavonoids and phenolic compounds along with different anti-cancer drugs, offering novel insights into their potential use in cancer treatment. The anti-cancer properties of various drugs, including doxorubicin, paclitaxel, 5-fluorouracil, and tamoxifen, are enhanced by flavonoids such as luteolin, quercetin, genistein, curcumin, and naringenin. These combinations target numerous molecular pathways, including angiogenesis, cell cycle control, apoptosis, and metastasis inhibition, that are implicated in the growth of cancer. In particular, luteolin and tamoxifen together inhibit Ras expression, which causes tamoxifen-resistant breast cancer cells to undergo apoptosis [188]. By increasing pro-apoptotic proteins like Bax and reducing anti-apoptotic proteins like Bcl-2, quercetin has also been reported in MDA-MB-231 and MCF-7 cell lines to increase the cytotoxicity of docetaxel and 5-fluorouracil, thus enhancing their therapeutic properties [189,190].

Furthermore, research has shown that genistein, an isoflavonoid, increases the cytotoxicity of treatments like tamoxifen and cisplatin and overcomes drug resistance in breast cancer models [191,192]. Another commonly used natural phenol, curcumin, was recently studied for the possible increase in the potency of drugs, including paclitaxel and doxorubicin. Curcumin has been proven in various studies to stimulate apoptosis, reduce the AKT/mTOR pathway, and initiate cell cycle arrest, thus reducing cancer cell proliferation [193,194,195,196]. Interestingly, in vivo studies show that curcumin may also synergize with chemotherapy treatments to decrease tumour development, improve the overall therapeutic outcome, and reduce the adverse effects of chemotherapeutic medications [193,197,198]. Likewise, natural substances like naringenin and naringin have demonstrated a remarkable potential to overcome chemoresistance and boost the anticancer properties of doxorubicin and 5-fluorouracil. Naringenin has been demonstrated to boost caspase activity, enhance the Bax/Bcl-2 ratio, and elevate cytokine production in several breast cancer models, hence stimulating therapeutic efficacy when combined with chemotherapeutic drugs [199,200]. These results emphasize the significance of flavonoids and phenolic substances in combination treatments for breast cancer. Their capacity to alter critical cancer-related processes, such as apoptosis, oxidative stress, and drug resistance processes, provides a viable technique for increasing the efficiency of conventional chemotherapy. The increasing number of findings shows that further clinical trials on these combination approaches may provide a new route for more potent, customized cancer therapies (Table 4).

The combined use of flavonoids with synthetic anti-cancer medicines indicates great potential in increasing treatment results via several mechanisms. Luteolin synergizes with tamoxifen to reduce Ras expression and initiate apoptosis in tamoxifen-resistant breast cancer cells [188]. Additionally, its combination with doxorubicin and paclitaxel highlights decreased cell survival and downregulated BCL-2 anti-apoptotic marker [201,202]. Quercetin, another important flavonoid, boosts the efficiency of docetaxel and letrozole by raising pro-apoptotic markers, including BAX and p53, while inhibiting proteins associated with cancer cell viability and migration, including STAT3, AKT, and MMPs [189,203]. Co-administration of curcumin with medications including docetaxel, sorafenib, and carboplatin underlines its potential in altering apoptotic pathways, ROS-induced DNA damage, and the tumor microenvironment, notably in triple-negative breast cancer (TNBC). Its anti-inflammatory and anti-metastatic capabilities, obtained by lowering COX-2 pathways and vimentin phases, further underscore its promise in combinatorial therapy [193,194,198]. Certain flavonoids, including naringenin and hesperidin, boost the efficiency of medications like doxorubicin and tamoxifen, as indicated by elevated pro-apoptotic gene expression and decreased tumor growth indicators in both in vitro and in vivo investigations [215,226]. Besides, the novel chemicals like xanthohumol and garcinol exhibit potential benefits in overcoming drug resistance and decreasing metastasis. In particular, xanthohumol efficiently reverses doxorubicin resistance by decreasing STAT3 and EGFR signaling, whereas garcinol reduces paclitaxel-induced pre-metastatic signaling via NF-κB/Twist1 pathways [212,214]. Furthermore, proanthocyanidins and mangiferin improve the action of 5-fluorouracil and doxorubicin, respectively, by boosting ROS generation, triggering cell cycle arrest, and reducing drug resistance proteins such as P-gp [220]. This study emphasizes the potential of flavonoid-based combinations to not only enhance treatment effectiveness but also reduce drug resistance in breast cancer, providing a strong case for additional clinical trials.

## 6. Advancement in Flavonoid Nano-Formulations and Codelivery Strategies in Breast Cancer Prevention

**Figure 4:**
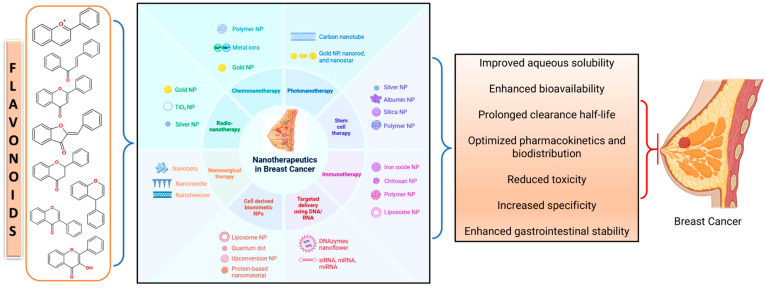
Flavonoid-based nanotherapeutic applications in breast cancer treatment.

The application of flavonoids in nanoparticle-derived drug delivery systems (DDSs) facilitates in situ targeting of cancer tissues, notably increasing drug concentration at the site of cancer while minimizing systemic toxicity, avoiding vascular injury, and lowering the extent of drug administration [231]. The anti-cancer efficacy of flavonoid-based nanotechnology is linked to various mechanisms, such as the stimulation of caspase enzymes, the introduction of cellular arrest, the decrease in cancer vascularization, the prevention of tumour cell proliferation and dissemination, the introduction of mitochondrial dysfunction, and apoptosis [232] (Figure 4). Different nanotechnologies, including phytosomes, liposomes, solid-lipid nanoparticles, nanocapsules, nanoemulsions, polymeric nanoparticles, lipid-based carriers, and metal-based nanoparticles, have been investigated for encapsulating polyphenols to improve their therapeutic efficacy in cancer therapy [233] (Table 5).

Polymeric nanoformulations, notably quercetin-loaded vehicles containing hyaluronic acid (230–480 nm) [234], chitosan (<200 nm) [235], and MPEG-PLA (155 ± 3 nm) [236], have demonstrated effectiveness in tumour suppression through the passive increased permeability and retention (EPR) effect and selective targeting via CD44 receptors. Moreover, mesoporous silica-based quercetin nanoparticles (<200 nm) regulate AKT and Bax signaling pathways to trigger apoptosis and cell cycle arrest [237], whereas EGCG-PEG nanoparticles (140–182 nm) augment tumor suppression by elevating p21, PTEN, and Bax expression while downregulating oncogenic markers such as p-AKT and Cyclin D1 [238]. Fisetin-PLA nanoparticles (225 ± 4 nm) have been documented to enhance bioavailability and exhibit anticancer properties [239]. Lipid–polymer hybrid nanoparticles, exemplified by quercetin–mycophenolic acid formulations (135–175 nm), enhance pharmacokinetics by diminishing first-pass metabolism and improving synergistic pharmacodynamics [240]. On the other hand, solid lipid nanoparticles containing EGCG with Bombesin (164 ± 2 nm) exhibit apoptotic actions by limiting nutrition access to cancer cells, thereby inhibiting migration and angiogenesis [241]. Moreover, metallic nanoparticles, such as gold nanoparticles functionalized with quercetin (5 nm) and AgFeO2 (19 nm), have produced anti-metastatic characteristics by obstructing epithelial–mesenchymal transition (EMT) and enhancing photodynamic treatment under ultraviolet light [242]. Hesperidin-encapsulated gold nanoparticles (40 nm) have also shown potential in regulating inflammatory cytokine release [244], while silver nanoparticles containing apigenin (94 nm) promote caspase-3-mediated apoptosis, thus increasing cytotoxicity against tumour cells [245]. Notably, utilising polymeric, lipidic, and metallic nanocarrier approaches can provide improved drug transport, tumour-targeted accumulation, and activation of apoptosis, presenting them as promising candidates for subsequent clinical application.

Contextually, phytosomes and liposomes are widely used in the market owing to their efficacy in medication delivery. Here, phytosomes, created by the electrostatic relationship between phospholipids and phytochemicals, have improved bioavailability of natural compounds. The Phytosome^®^ technology, created by an Italian pharmaceutical firm, has shown efficacy in enhancing oral absorption of poorly absorbed compounds [246]. When compared to their unencapsulated counterparts, lipid-based nanoformulations containing flavonoids, including myricetin, EGCG, and quercetin, have stronger anti-cancer action. Moreover, when compared to non-conjugated compositions, EGCG-loaded nanoparticles conjugated with the gastrin-releasing peptide receptor (GRPR)-specific peptide bombesin demonstrated enhanced therapeutic potency and anti-migration ability in in vitro MDA-MB-231 and B16F10 cancer cell lines and in vivo female C57/BL6 mice. [241]. Intriguingly, metallic nanoparticles containing flavonoids, such as EGCG, quercetin, hesperidin, and apigenin, have also received interest owing to their improved therapeutic properties. For example, hesperidin-loaded gold nanoparticles (Hsp-AuNPs) were demonstrated to promote cytotoxicity in MDA-MB-231 cells in vitro, whereas toxicity studies in BALB/c mice indicated no obvious harm or histological alterations at dosages of 20 mg/kg and 200 mg/kg [244]. Additionally, new research on naringenin coupled with cyclophosphamide in breast cancer cells has emphasized their anti-proliferative properties, especially using the activation of STAT-3 and JAK-2 pathways. This combined therapy exhibits the capacity to prevent the uncontrolled proliferation of cancer cells, highlighting its potential as an effective therapeutic agent for breast cancer chemotherapy [247]. Additionally, nanoemulsions have been suggested as a viable delivery strategy to boost the absorption and solubility of naringenin [248]. Nano-naringenin significantly blocks both PI3K and MAPK pathways, and conserves ER-alpha in the cell membrane, therefore lowering proliferation in tamoxifen-resistant breast cancer cells (Tam-R) [249]. Furthermore, silibinin-loaded nanomaterials (SLNs) have exhibited targeted cancer development, revealing higher inhibitory properties on MDA-MB-231 cells than with pure silibinin, mainly caused by the inhibition of Snail and MMP [250,251,252]. Genistein, another flavonoid with breast cancer-preventive characteristics, has been demonstrated to stop MCF-7 cell development in the G2/M phase while lowering proliferation in the S phase [253]. A unique genistein-loaded PEGylated silica hybrid nanoparticle has also been produced utilizing a basic aqueous dispersion process, which indicated increased breast cancer therapy effectiveness, as validated by infrared examination exhibiting packed encapsulation of genistein [254,255]. The mechanisms of action and different nanotechnologies of flavonoids and their co-delivery in breast cancer therapy are explained extensively in Table 6.

The use of flavonoid-based nano-formulations in breast cancer treatment is an effective approach for enhancing drug solubility, stability, and targeted delivery. Liposome-based systems have been extensively used, with quercetin co-encapsulated with medicines including vincristine, doxorubicin, and Adriamycin exhibiting synergistic effects, decreased tumor progression, and controlled release [256,257,258]. PEGylated liposomes and polymeric nanoparticles, particularly those that integrate quercetin with tamoxifen, successfully attenuate drug resistance by regulating pathways including P-glycoprotein and Nrf2, resulting in increased oxidative stress and apoptosis [273].

Furthermore, nano-formulations containing silibinin, EGCG, curcumin, and chrysin exhibit several therapeutic actions. This includes inducing apoptosis, inhibiting angiogenesis, and suppressing tumor-promoting pathways like PI3K/AKT/mTOR [261,267,270,282]. Likewise, lipid-based systems, especially lipid nanoparticles and nano-emulsions, enhance flavonoid stability and cellular absorption while decreasing toxicity. Additionally, polymeric micelles and gold nanoparticles have favorable effects on cancer-specific targets, such as DNA–topoisomerase complexes and apoptotic genes [285,286]. Additionally, combinations such as baicalein–paclitaxel or rapamycin–curcumin exhibit substantial tumor suppression and prolonged drug release, highlighting the significance of dual-loaded systems [272,279]. Hence, all these research reports focus on important issues in the treatment of breast cancer by highlighting the critical role that flavonoid-based nano-carriers provide to achieve improved therapeutic efficacy, decreased multidrug resistance, and targeted cancer cell death.

Various methods of production are used for the development of flavonoid-based nano-formulations [287]. Nanoprecipitation is a prevalent method that depends on the fast combination of a polymer or lipid solution with a non-solvent, which results in the natural production of nanoparticles with a homogeneous size distribution [288]. Thin-film hydration is a widely used technique in which lipids are dissolved in organic solvents, evaporated to form a thin film and then hydrated with an aqueous phase to produce liposomes or analogous carriers [289,290]. Solvent evaporation requires emulsifying a drug–polymer combination in an aqueous phase and then removing the solvent under decreased pressure to produce stable nanoparticles [291]. Sonication-assisted methods are often used to decrease particle size and enhance uniformity. They are typically utilised as a subsequent step to refine liposomes or other vesicular systems [292,293]. Recent investigations into microfluidic techniques have focused on attaining rigorous control over particle sizes and consistency, where green synthesis techniques prioritise the use of biocompatible solvents and natural stabilisers to improve safety and sustainability [294]. Optimization of these nano-formulations is necessary to obtain optimal therapeutic effects. Key factors involve the lipid-to-drug or polymer-to-drug ratio, which affects encapsulation efficiency and drug release [295,296]. Surfactant concentration and type influence particle stability and prevent aggregation. Process parameters, including hydration temperature, stirring velocity, and sonication duration, are critical in regulating particle size and polydispersity index [297,298]. Systematic optimising of these factors promotes drug loading, stability, and bioavailability, thus enhancing the efficacy of flavonoid-based nano-formulations in breast cancer therapy [299,300].

## 7. Safety, Toxicity, and Regulatory Aspects of Flavonoid-Based Nanoformulations

Flavonoid-based nanoformulations provide a potential technique to boost the bioavailability and therapeutic effectiveness of flavonoids in cancer therapy. However, their inclusion into nanocarriers raises significant safety and toxicity problems that need thorough consideration [301,302]. While flavonoids themselves are typically harmless, encapsulation in nanoparticles may change their pharmacokinetics and interactions with biological systems [303]. Nanotechnology-based carriers like lipid-based nanoparticles and polymeric micelles may increase stability and allow controlled release, thus decreasing systemic toxicity. However, particle size, surface charge, and composition might alter cellular absorption and distribution, which can lead to harmful effects in non-target tissues [304,305].

Additionally, nanoparticles may accumulate in organs, notably the liver, spleen, and kidneys. This is a potential concern of organ-specific damage, such as hepatotoxicity and nephrotoxicity [306,307,308]. Oxidative stress and inflammation in various organs have been identified in select investigations, stressing the necessity for long-term in vivo study. Interaction with the immune system is another important factor. Nanomaterials can induce immunological activation and pro-inflammatory responses, which may result in hypersensitivity or chronic inflammation [309,310]. Surface modifications, such as PEGylation, may decrease immunogenicity, but their long-term safety is yet to be completely understood [311,312].

The regulatory framework for flavonoid-based nanoformulations is challenging and varied between areas, reflecting the particular issues faced by nanomedicines. The US Food and Drug Administration offers recommendations for drug products incorporating nanomaterials, highlighting the necessity for rigorous assessment of physicochemical attributes, pharmacokinetics, and possible toxicity. Risk assessment and mitigation measures are needed to assure the safety, quality, and effectiveness of these formulations [313,314]. Additionally, the European Medicines Agency (EMA) has produced recommendations that concentrate on the quality, safety, and effectiveness of nanomedicines. The EMA underlines the necessity of analysing nanomaterial characteristics, biodistribution, and pharmacokinetics, supporting preclinical and clinical investigation of immunogenicity and organ-specific toxicity [315,316,317]. At the worldwide level, organizations such as the International Council for Harmonisation are attempting to unify regulatory standards. Efforts are ongoing to standardize procedures for nanomaterial characterisation and safety evaluation, facilitating worldwide research, approval, and clinical translation of nanomedicines, including flavonoid-based formulations [318].

## 8. Conclusion and Future Perspectives: Advancing Flavonoid Research in Cancer Therapy

Flavonoids, with their various pharmacological effects, have been identified as potential medicines in the prevention and treatment of breast cancer. Their capacity to cause apoptosis, suppress cell growth, and affect several biological pathways makes them viable options in the production of innovative anti-cancer medicines. However, despite their enormous medicinal potential, the clinical application of flavonoids has been limited by difficulties such as limited bioavailability, poor solubility, and quick metabolism. The development of nanotechnology has created new opportunities for addressing these limitations, since nanoparticle-based drug delivery systems have exhibited increased stability, solubility, and specific therapeutic administration of flavonoids. While multiple scientific studies have shown the anti-cancer potential of flavonoid-based treatments, additional clinical studies are required to assess their safety, efficacy, and appropriate dose in individuals. The combined administration of flavonoids with conventional chemotherapeutic drugs also has considerable promise, but understanding the synergistic advantages and reducing possible toxicities remains a crucial topic for future studies.

Current studies on flavonoid-based breast cancer therapy should advance beyond single-agent trials to extensively investigate combination approaches with existing chemotherapeutics, hormonal drugs, and targeted therapies. These strategies may overcome drug resistance, improve therapeutic effectiveness, and reduce toxicity. However, careful optimization of dosage, sequencing, and delivery methods is required to attain clinically significant results. Nanotechnology will remain an essential facilitator in this domain. Development in co-delivery systems, stimuli-responsive nanocarriers, and tailored nanomedicine techniques may greatly improve the pharmacokinetic limitations of flavonoids and allow precise targeting of tumor sites. Simultaneous synthesis of novel flavonoid combinations with enhanced stability and bioavailability is expected to provide new opportunities for therapeutic applications.

However, effective clinical translation will necessitate a deeper attention to safety and toxicity. Long-term in vivo studies are required to address problems such as organ-specific accumulation, immunogenicity, and chronic toxicity. Employing sophisticated analytical methods for nanoparticle characterization and continuous evaluation in biological systems will be vital to bridge the gap between laboratory effectiveness and patient safety. Furthermore, the regulatory framework must adapt to scientific advancement. Collaborative efforts with authorities such as the FDA, EMA, and ICH are vital for creating harmonized criteria for nanomedicine approval, including consistent techniques for characterization, toxicity assessment, and pharmacokinetic evaluation. Only by using the convergence of pharmacological innovation, nanotechnology, toxicology, and regulatory science can flavonoid-based nanoformulations proceed from promising laboratory results to safe and effective clinical therapy for breast cancer.

## Figures and Tables

**Table 2:** Clinical trials of some flavonoids in combination with anti-cancer drugs. Adapted from Vachetta et al. [22] under the Creative Commons CC BY 4.0 license.

Flavonoid	Clinical Trial	Objective of the Study	Clinical Trail Phase	NCT Number	Current Status/Year
Genistein	Biomarker Assays for a Phase 2 Trial of gemcitabine and genistein in Women with Metastatic Breast Cancer	Explore a target outcome among women with breast cancer at stage IV. The treatment included genistein and gemcitabine hydrochloride.	Phase 2	NCT00244933	Completed/2023
	The Role of Genistein in Breast Cancer Prevention in Women at elevated risk	Evaluate the role of genistein on the growth of breast epithelial cells isolated from women at elevated risk for breast cancer using a small-needle approach.	Phase 2	NCT00290758	Completed/2017
	The Function of Genistein in the Prevention of Breast and Endometrial Cancer in Healthy Postmenopausal Women	Assess the efficacy of genistein in reducing DNA damage and facilitating apoptosis for preventing the development of breast and endometrial cancer in normal postmenopausal women.	Phase 1	NCT00099008	Completed/2013
AFP464, Aminoflavone	Examination of AFP464 and Faslodex in Oestrogen Receptor-Positive Breast Carcinoma	The response to clinical advantage	Phase 2	NCT01233947	Terminated/2012
	AFP464 in the Management of Patients with Solid tumours That Are Metastatic or Refractory and Unsuitable for Surgical Removal	Examine the disadvantages and ideal dose of AFP464 for patients with solid tumours that are metastatic or resistant to radiation therapy.	Phase 1	NCT00348699	Completed/2014
ME-344 (synthetic molecule having isoflavan ring structure)	ME-344 (57) has anti-angiogenic properties that change mitochondrial metabolism in early HER2-negative breast cancer.	When the mitochondrial phenotype has been produced, determine if adding ME-344 (57) to anti-angiogenic drugs improves anti-tumor action.	Early Phase 1	NCT02806817	Completed/2019
Plant extracts	The Function of Plant-Based Dietary Supplements in Intravenous Chemotherapy	Analyze the quantitative effects of dietary supplement consumption on breast cancer patients receiving intravenous infusions of neo-adjuvant and/or adjuvant chemotherapy.	-	NCT03959618	Completed/2021
	Effect of Watercress Consumption on Cancer Patient Results: A Longitudinal Analysis	Examine the possible advantages of therapeutic diets enhanced with watercress-derived nutraceuticals, giving particular attention to how they affect DNA damage and their greater impact on the prognosis of diseases worldwide.	Phase 3	NCT02468882	Unknown status/2015
S-Equol	A Pre-Surgical Clinical Trial Assessing the Efficacy of S-Equol Therapy in Women with Triple-Negative Breast Cancer (TNBC)	To assess the effectiveness of the ERβ agonist S-equol in preventing triple-negative breast cancer (TNBC) cells from proliferating.	Phase 1	NCT02352025	Completed/2020
Flavonoids	Effect of Amino Acids and Flavonoids Containing FSMP on Chemotherapy Toxicity, Nutritional Status and Quality of life in Breast Cancer patients	To assess whether an amino acid- and flavonoid-based FSMP with nutritional counseling improves nutritional status, quality of life, and reduces chemotherapy toxicity in breast cancer patients compared to counseling alone.	Not Applicable	NCT05968677	Recruiting/2025
Hesperidin and Diosmin	The Effect of Oral Administration of Hesperidin and Diosmin in Reducing Paclitaxel-induced Peripheral Neuropathy in Breast Cancer Patients	To evaluate the neuroprotective potential of oral hesperidin and diosmin in reducing paclitaxel-induced peripheral neuropathy in breast cancer patients.	Phase 3	NCT06811220	Recruiting/2025
Quercetin	Dasatinib Combined With Quercetin to Reverse Chemo Resistance in Triple-Negative Breast Cancer	To evaluate the efficacy and safety of dasatinib and quercetin in combination with chemotherapy in mTNBC patients who have progressed on prior chemotherapy.	Phase 2	NCT06355037	Recruiting/2024

**Table 3:** Patents on Flavonoids in Combination with Anti-Cancer Drugs for Breast Cancer Treatment.

Flavonoids (s)	Synthetic Drug(s)/Combination Agents	Key Patent Claims & Features	Status	Patent Number/Title
Isoflavonoids, hydroxyphenyl flavonoids	Kinase inhibitors (nintedanib, dovitinib, regorafenib)	Combination therapies for breast cancer using flavonoids and kinase inhibitors; various dosage forms including oral/IV/topical	Patent granted	ES2877712T3
Luteolin, quercetin, kaempferol	Docetaxel, cisplatin, enzalutamide, radiotherapy	Fixed molar ratio flavonoid blends combined with synthetic chemo/hormonal/radiotherapy; oral and parenteral forms	Patent published	US20170087125A1
Catechins, anthocyanidins, and isoflavones.	Chemotherapeutic agents	Compositions including flavonoids plus chemotherapeutics to enhance anti-cancer effects	Patent granted	US20120213842A1
Flavonoids/antioxidants	Cyclin-dependent kinase (CDK) inhibitors	Therapeutic combinations of flavonoids with CDK inhibitors targeting cancer progression	Patent granted	US10555931B2
Anti-oncogenic flavonoids	Cisplatin, doxorubicin	Novel anti-oncogenic phytochemical combinations with synthetic drugs for cancer treatment	Patent granted	US20240009163A1
Flavonoid mixtures (quercetin, vitamin C, Blueberry Extract)	Methantheline	Flavonoid compositions combined with chemotherapy drugs; methods of use patent	Patent published	WO2017053583A1

**Table 4:** The combination effect of flavonoids with synthetic drugs.

Flavonoids/Phenolic Compounds	Anti-Cancer Drug	In Vitro/In Vivo	Cancer Cell Lines/Animal Study	Effect	References
Luteolin (Flavone)	Tamoxifen	In vitro	MCF-7	Suppresses Ras expression to cause apoptosis in tamoxifen-resistant ER-positive breast cancer cells.	[188]
	Doxorubicin	In vitro	MCF-7	The viability of cells decreased.	[201]
	Paclitaxel	In vitro	MDA-MB-231	Downregulation of BCL-2, mRNA expression	[202]
Quercetin (Flavone)	Docetaxel	In vitro	MDA-MB-231	Significantly higher BAX levels and upregulated p53 are coupled with decreased expression of the proteins BCL2, pERK1/2, AKT, and STAT3.	[189]
	5-fluorouracil	In vitro	MDA-MB-231	Decreased MMP-2 and MMP-9 gene expression levels and a major decline in migration rate.	[190]
		In vitro	MCF-7	Raising p53, caspase-9 activity, and Bax expression while lowering Bcl2 expression	[91]
	Letrozole	In vitro	MCF-7	Caused apoptosis produced by the mitochondria and suppressed cellular growth.	[203]
			MDA-MB-231		
	Doxorubicin	In vitro	Doxorubicin MCF-7-resistant	Decreased levels of important genes, such as SNAI2, PLAU, and CSF1, which reverses doxorubicin resistance in breast cancer cells.	[204]
			MDA-MB-231	The MMP-2 and MMP-9 genes showed decreased migration and expression.	[205]
Genistein (Isoflavone)	Exemestane	In vitro	MCF-7	Enhanced cytotoxicity	[206]
	Tamoxifen	In vitro	MCF-7	Potential to enhance TAM therapy	[191]
	Cisplatin	In vitro	Ovariectomized nude mouse breast cancer xenograft model	Enhanced the development of apoptosis and inhibited cell growth.	[192]
	ERB-041	In vitro	CMT-U27; CF41.Mg	Downregulated the expression of ERα, which in turn decreased the regulation of the PI3K/AKT pathway.	[207]
	Centchroman	In vitro	MDA-MB-468 MDA-MB-231 MCF-7	Synergistic anti-cancer potential	[208]
Curcumin (Natural phenol)	Docetaxel	In vitro	MCF-7	Maximum rates of cytotoxicity, cell apoptosis induction, and cellular uptake	[193]
	Sorafenib	In vitro	MCF-7	Apoptotic cell death is induced when vimentin, IL-6, STAT3, and MMP-9 levels are decreased and E-cadherin protein expression is increased.	[194]
	Doxorubicin	In vitro	MDA-MB-231	Indicated that the AKT/mTOR pathway is being suppressed.	[195]
		In vitro	MDA-MB-231 MCF-7	G0/G1 and S-phase cell cycle arrest were associated with enhanced mRNA expressions of the TP53, BRCA1, BRCA2, ATM, and CHEK2 genes (Ct-value).	[196]
	Paclitaxel	In vitro	MCF-7 MDA-MB-231	Curcumin decreased capacity of M2 TAMs to generate chemoresistance.	[197]
	Apatinib	In vitro	MCF-7	Reduced proliferation and survival	[209]
	Celecoxib	In vitro	MDA-MB-231	Suppressing the COX-2 pathways.	[210]
	5-Fluorouracil	In vitro	MCF-7	Pro-apoptotic, anti-metastatic, and anti-proliferative.	[211]
	Carboplatin	In vitro	CAL-51, CAL-51-R, MDA-MB-231 cells	By enhancing ROS-induced DNA damage, curcumin makes TNBC more susceptible to the anti-cancer effects of carboplatin, offering an efficient combined therapy approach for TNBC.	[198]
Xanthohumol (Prenylflavonoid)	Doxorubicin	In vitro	Doxorubicin-resistant breast cancer cells MCF-7/ADR	Decreased the viability of tumor cells, caused apoptosis, slowed the cell cycle, improved the effects of doxorubicin, decreased stemness, elevated c-H2AX, inhibited STAT3 and EGFR, decreased Bcl-2 and pro-caspase 3, and improved Bax expression.	[212]
			Doxorubicin-resistant breast cancer cells MCF-7/ADR	Reduced protein expression via inhibiting STAT3 and EGFR, which in turn controlled apoptosis resistance; reduced the expression of MDR1 but not BCRP.	[213]
Garcinol (Polyphenol)	Paclitaxel	In vivo	4T1-Luc female Balb/c mice (5–6-weeks-old)	Downregulation of caspase-3, iPLA2, Cyclin A2, Cyclin B1, Cdc25A, Cdc2, Bcl-2, and COX-2, inhibition of paclitaxel-induced NF-κB/Twist1-regulated premetastatic signalling, and lower MMP-2 and MMP-9 activity were observed.	[214]
Naringenin	Cyclophosphamide	In vitro	MDA-MB-231	BAX expression increased whereas Bcl-2 expression decreased. Caspases 3 and 9 were stimulated.	[199]
	Doxorubicin	In vitro	Breast cancer mouse model	Combination therapy improves anticancer activity in vivo, reducing tumour volume and weight.	[215]
		In vitro and In vivo	MDA-MB-231 and 4T1 cells Xenograft mouse model	Increased cytokines (TNF-α and IL-1β) and decreased dose-related body weight loss	[200]
			T47D and MCF-7 cells	In p53-deficient T47D cells, naringenin and hesperidin increased doxorubicin-induced G2/M arrest through a p53-independent pathway. whereas, in p53 wild-type MCF-7 cells, they inhibited G2/M arrest, showing that their anticancer activity is not dependent on cell cycle arrest.	[216]
Naringin	Capecitabine	In vitro	MCF-7 and SK-BR-3	Elevation in the Bax/Bcl-2 ratio	[217]
	Doxorubicin	In vitro	MCF-7	Significantly raised the level of Bax while inhibiting the expression of STAT3, JAK1, Bcl-2, Survivin, and VEGF.	[218]
	5-Fluorouracil	In vitro	MDA-MB-231	Reduced cytological toxicity of 5-Fluorouracil	[219]
Mangiferin (Polyphenolic C-glycoside)	Doxorubicin	In vitro	MCF-7 had already received short-term Doxorubicin	Suppressed P-gp expression	[220]
Proanthocyanidins (Polyphenols)	Histone deacetylase inhibitor Chidamide	In vitro	T47D and MDA-MB-231	Suppress the growth and proliferation, while promoting apoptosis	[221]
	5-Fluorouracil		MDA-MB-231	Cell viability and migration declined, apoptosis was induced, cell cycle was arrested in the G2/M phase, generated ROS was increased, mitochondrial membrane potential reduced, the Bax/Bcl-2 ratio was increased, caspase-3 was cleaved, and there was synergistic cytotoxicity with 5-fluorouracil.	[222]
Apigenin	Doxorubicin	In vitro	DOX resistant MCF-7 cells and MCF-7R	Reduced the phosphorylation and induction of JAK2 and STAT3 proteins.	[106]
			MCF-7	Interact with DNA, thus blocking the transcription process	[223]
	Vorinostat		MDA-MB-231	Increased HAT activity while suppressing DNMT and HDAC enzymatic activity.	[224]
Kaempferol	Docetaxel	In vitro	MCF-7	Anti-CSCs agent (decreasing the markers linked to CSCs, such as aldh1a1 and abcb1)	[225]
Hesperidin	Doxorubicin	In vitro	Hela Cell Line	Enhanced the expression of Bax and reduced the level of Bcl-2	[226]
			MCF-7/HER2 cells	Reduced HER2, Rac1, MMP9 expression, apoptosis, and cell migration after inducing cell cycle arrest	[227]
			MCF-7 resistant doxorubicin cells	Reduced level of Pgp expressions	[228]
			4T1	Inhibition of Rac-1 and metalloproteinase-9 expression	[229]
	Tamoxifen	In vivo	xerographic MCF-7 injected rats	Apoptotic genes (Bax, Casp3) are upregulated, while antiapoptotic genes (Bcl-2) and angiogenesis genes VEGF are downregulated.	[230]

**Table 5:** Flavonoid-loaded nano-formulation for breast cancer treatment.

Nano-Formulation	Flavonoid/Phenolic Compounds	Size (nm)	Effect	References
Polymeric nanoformulation	Quercetinhyaluronic acid	230–480 nm	Prevent tumour development in tumour-developing mice using the passive EPR effect selective targeting mediated by CD44 receptor.	[234]
	Quercetin–chitosan	<200	Elevated serum SOD levels	[235]
	Quercetin–MPEG-PLA	155 ± 3	Induction of cell apoptosis	[236]
	Quercetin–Mesoporous silica	<200	Apoptosis and cell cycle arrest by controlling the AKT and Bax signalling pathways-1.	[237]
	EGCG-PEG	140 ± 7 to 182 ± 13	Increased expression of p21, PTEN, and Bax, while inhibition of p-AKT, p-PDK1, Bcl-2 and Cyclin D1, reduced cancer cell migration.	[238]
	Fisetin–PLA	225 ± 4	Enhance the bioavailability and anti-cancer activity of fisetin.	[239]
Lipid –polymer nanoparticles	Quercetin–Mycophenolic acid	135 ± 10 to 175 ± 30	Improved pharmacokinetics/pharmacodynamics, reduced first-pass metabolism and displayed additive/synergistic pharmacodynamics.	[240]
Solid-lipid nanoparticles	EGCG–Bombesin	164 ± 2	Both intrinsic and extrinsic routes can cause apoptosis, which reduces the amount of nutrients that cancer cells receive and prevents migration and angiogenesis.	[241]
Gold Nanoparticle	Quercetin–AgFeO2	19	Under UV light, photodynamic therapy reduces cell growth.	[242]
	Quercetin	5	Decreased expression of Vimentin, N-cadherin, Snail, Slug, Twist, MMP-2, MMP-9, p-EGFR, VEGFR-2, p-PI3K, Akt, and p-GSK3β, along with increased expression of E-cadherin protein, substantially decreased epithelial–mesenchymal transition, angiogenesis, and metastasis.	[243]
	Hesperidin	40	prevent cytokine secretion, including IL-1β, IL-6, IL-8, NO, and TNF-α, causing a rise in ROS.	[244]
Silver Nanoparticles	Apigenin	94	Enhance caspase-3 protein, causing apoptosis in tumour cells.	[245]

**Table 6:** Codelivery systems of flavonoid nano-formulation for breast cancer treatment.

Nano-Formulation	Flavonoid/Phenolic Compounds	Size (nm)	Effect	References
Liposomes	Quercetin and vincristine	130–200	Co-encapsulated medicines improved quercetin solubility, resulting in synergistic effects and controlled release.	[256]
	Quercetin and vincristine	130	Successful reduction of tumour development in the JIMT-1 patient breast carcinoma xenograft model	[257]
PEGylated Liposomes	Quercetin–Adriamycin (AMD) and doxorubicin	85	The combination prevents AMD-induced myelosuppression, elevates white blood cell levels, decreases myocardial cell death due to AMD, and promotes toxicity against resistant tumour cells.	[258]
Liposomes	Quercetin and doxorubicin (DOX)	85 ± 2	Reduced levels of Nrf2 and its related cytoprotective enzymes, including NQO1 and the drug transporter MRP1, is linked to apoptosis induction.	[259]
PLGA-Casein Nanoparticle	EGCG and paclitaxel (PTX)	190 ± 12	PTX triggered apoptosis while also suppressing essential genes required for tumour survival.	[260]
Lipid Nanoformulation	Silibinin–TPGS	45	Reduced levels of MMP-9 and Snails	[261]
Albumin based nanoparticles	Epicatechin and morin	170 ± 6	Prevent the successful proliferation of cancer cells, leading to cell death.	[262]
Nanoparticles based on polyethylene glycol.	Quercetin and Nickel	50–700	Reduced mitochondrial membrane potential and increased oxidative stress brought on by an excess of ROS.	[263]
Zein-lactoferrin micelles (Lipid nanoparticles)	Rapamycin and wagonin	277	Reduced levels of the PI3K-AKT and MAPK pathways, together with anti-angiogenic effects.	[264]
AEEAA-PEG-PCL Nanoparticle	Silibinin and IPI-549	35–37	Inhibition of collagen production, angiogenesis, and anti-fibrotic effects in cancerous tissue	[265]
pW lipid Nano-particles	Silibinin and cryptotanshinone	250	Suppress CD31, TGF-β1, and MMP-9.	[266]
Polymeric Nanoparticle	Chyrisin and methotrexate	198	Enhanced disruption and cell wall shrinking causes apoptosis.	[267]
	Chyrisin and curcumin	400–500	Upregulation of Cyclin D1, hTERT, Bax/Bcl-2, p53, caspase-3 & 7.	[268]
Zein protein Nanocarrier	EGCG and piperine	34–80	The drug is beneficial in NPS because of its higher cellular intake and extended release.	[269]
Gold Nanoparticles	EGCG and citrate	25	It efficiently controls NF-κB, increasing apoptotic proteins like caspase-3, caspase-7, and Bax and decreasing anti-apoptotic proteins like Bcl2.	[270]
Polymeric Nanoparticles	EGCG and curcumin and alpha-tocopheryl succinate	200–300	Modified with transferrin and folate, two ligands unique to cancer that support antiproliferative activity.	[271]
Nanoemulsion	Baicalein–paclitaxel	171 ± 6.2	Decreased glutathione synthesis, and initiated apoptosis	[272]
Polymeric Nanoparticles	Quercetin–tamoxifen	>200	Reduced MMP-2 and MMP-9, enhanced oxidative stress, and decreased GSH caused by lipid peroxidation	[273]
Nanoemulsion	Naringenin–tamoxifen	<701	Reduced P-gp efflux leads to increased anticancer activity.	[274]
Nanomicelles	EGCG–Herceptin1	100–500	Extended blood half-life and decreased cancer cell growth.	[275]
Polymeric nanoparticles	Quercetin–doxorubicin	105	P-glycoprotein expression and function are diminished.	[276]
Polymeric Nanoparticles	Quercetin–topotecan	<200	Cell death occurs when there are significant modifications in the endoplasmic reticulum, mitochondria, and cell wall.	[277]
Nanofibers	Quercetin and tamoxifen citrate	Uniform size	The slow process of tumour development	[278]
Polymeric Nanoparticles	Genistein–Chitosan–Tamoxifen	299.8	The slow process of tumor development	[191]
Nanomicelles	Docetaxel and Curcumin	~64	The greatest levels of cellular absorption, cytotoxicity, cell apoptosis induction, and reactive oxygen species (ROS) production	[193]
Liposomes	Rapamycin–curcumin	Uniform size	Target mTOR to inhibit breast cancer development.	[279]
nanocomposites	chitosan/agarose/graphene oxide/montmorillonite–curcumin		Extended release of curcumin to inhibit MCF7 breast cancer cells	[280]
Nanomicelles	Doxorubicin and Curcumin	187 ± 1.36	Activated apoptosis and reduced multidrug resistance (MDR) in breast cancer	[281]
Nanoparticles	curcumin/Ko143/PLGA	232.32 ± 10.60	Promote the intracellular level of the photosensitiser Curcumin (Cur) and improve photodynamic effectiveness by inhibiting the function of efflux pump.	[282]
Nanoparticles	Paclitaxel and curcumin	85.8 ± 0.21	Showed significant tumour suppression; prevent the formation of P-glycoprotein.	[283]
Niosome	Doxorubicin and curcumin	200	Regulation of PI3K/Akt/mTOR	[284]
Polymeric Micelles	Rutin/Benzoic Acids/Triazolofluoroquinolones	18 ± 2	Interaction with the DNA-Topoisomerase I enzyme complex.	[285]
Gold nanoparticle	Kaempferol and sorafenib	Uniform size	Enhanced apoptosis rate.	[286]

## Data Availability

The raw data supporting the conclusions of this article will be made available by the authors on request.
