# Correction: Uniyal et al. Flavonoid-Based Combination Therapies and Nano-Formulations: An Emerging Frontier in Breast Cancer Treatment. *Pharmaceuticals* 2025, *18*, 1486

**DOI:** 10.3390/ph19071021

**Published:** 2026-06-30

**Authors:** Priyanka Uniyal, Ansab Akhtar, Ravi Rawat

**Affiliations:** 1 Department of Pharmaceutical Sciences, School of Health Sciences and Technology, University of Petroleum and Energy Studies (UPES), Dehradun 248007, India; upiya25@gmail.com; 2 Neuroscience Center, School of Medicine, LSU Health, New Orleans, LA 70112, USA

**Missing Citation**

In the original publication [1], the following reference was not cited:

Vachetta, V.S.; Marder, M.; Troncoso, M.F.; Elola, M.T. Opportunities, Obstacles and Current Challenges of Flavonoids for Luminal and Triple-Negative Breast Cancer Therapy. *Eur. J. Med. Chem. Rep.* **2022**, *6*, 100077. https://doi.org/10.1016/j.ejmcr.2022.100077.

The citation has now been inserted in Figure 1 legend, and in the legends of Tables 1 and 2. The corrected legends appear below.

**Figure 1.** Classification of flavonoids and their chemical structures. Adapted from Vachetta et al. [22] under the Creative Commons CC BY 4.0 license.
**Table 1.** Therapeutic effects of flavonoids in breast cancer treatment. Adapted from Vachetta et al. [22] under the Creative Commons CC BY 4.0 license.
**Table 2.** Clinical trials of some flavonoids in combination with anti-cancer drugs. Adapted from Vachetta et al. [22] under the Creative Commons CC BY 4.0 license.

With this correction, the order of some references has been adjusted accordingly. The authors state that the scientific conclusions are unaffected. This correction was approved by the Academic Editor. The original publication has also been updated.

## References

[1] Uniyal P., Akhtar A., Rawat R. (2025). Flavonoid-Based Combination Therapies and Nano-Formulations: An Emerging Frontier in Breast Cancer Treatment. Pharmaceuticals.

